# Do Biofilm Formation and Interactions with Human Cells Explain the Clinical Success of *Acinetobacter baumannii*?

**DOI:** 10.1371/journal.pone.0010732

**Published:** 2010-05-20

**Authors:** Anna de Breij, Lenie Dijkshoorn, Ellen Lagendijk, Joke van der Meer, Abraham Koster, Guido Bloemberg, Ron Wolterbeek, Peterhans van den Broek, Peter Nibbering

**Affiliations:** 1 Department of Infectious Diseases, Leiden University Medical Centre, Leiden, The Netherlands; 2 Institute of Biology, Leiden University, Leiden, The Netherlands; 3 Department of Molecular Cell Biology, Section Electron Microscopy, Leiden University Medical Centre, Leiden, The Netherlands; 4 Institute of Medical Microbiology, University of Zurich, Zurich, Switzerland; 5 Department of Medical Statistics, Leiden University Medical Centre, Leiden, The Netherlands; Institut de Pharmacologie et de Biologie Structurale, France

## Abstract

**Background:**

The dramatic increase in antibiotic resistance and the recent manifestation in war trauma patients underscore the threat of *Acinetobacter baumannii* as a nosocomial pathogen. Despite numerous reports documenting its epidemicity, little is known about the pathogenicity of *A. baumannii*. The aim of this study was to obtain insight into the factors that might explain the clinical success of *A. baumannii*.

**Methodology/Principal Findings:**

We compared biofilm formation, adherence to and inflammatory cytokine induction by human cells for a large panel of well-described strains of *A. baumannii* and compared these features to that of other, clinically less relevant *Acinetobacter* species. Results revealed that biofilm formation and adherence to airway epithelial cells varied widely within the various species, but did not differ among the species. However, airway epithelial cells and cultured human macrophages produced significantly less inflammatory cytokines upon exposure to *A. baumannii* strains than to strains of *A. junii*, a species infrequently causing infection.

**Conclusion/Significance:**

The induction of a weak inflammatory response may provide a clue to the persistence of *A. baumannii* in patients.

## Introduction

With the recent description of *Acinetobacter bereziniae* (genomic species (gen. sp.) 10), *A. guillouiae* (gen. sp. 11) [1], *A. venetianus* [2] and *A. soli* [3], the genus *Acinetobacter* comprises 23 named species and 11 species with provisional designations. Of these, *A. baumannii* and the closely related *A.* gen. sp. 3 and 13TU are clinically the most relevant. Strains of these species have the ability to colonize and spread among critically ill hospitalized patients. Outbreaks of multidrug resistant *A. baumannii* strains have been observed worldwide [4], [5]. A striking manifestation is the dramatic increase in the frequency of imipenem resistant *Acinetobacter* isolates in US hospitals [6] and the recent occurrence of infection in severely injured soldiers during the conflicts in Iraq and Afghanistan [7]. Three major lineages of genetically highly related *A. baumannii* strains, designated European clone I, II and III, have been found to be frequently implicated in outbreaks [8], [9]. Other *Acinetobacter* species, such as *A. junii, A. johnsonii* and *A. lwoffii* that can frequently be found on the human skin are only incidentally involved in infection, which usually has a mild course [4]. This suggests differences in the pathogenic potential among *Acinetobacter* species.

The high prevalence of *A. baumannii* strains in the hospital in epidemic and endemic situations might be explained by several factors, including their resistance to antibiotics [10] and desiccation [11], their ability to form biofilms on medical devices [12], and to colonize skin and mucosal surfaces of vulnerable hosts [13], [14]. Adherence of bacteria to host cells is generally considered to be an essential initial step in the colonization process [15]. Once the primary colonizing bacteria have attached to a surface, microcolonies are formed after which bacteria may secrete exopolysaccharides resulting in a highly structured sessile microbial community, the biofilm [16]. Several studies have documented the ability of *A. baumannii* to adhere to epithelial cells and to form biofilms on glass and plastic surfaces [12], [17], [18]. Adherent bacteria can interact with cells of the host defense systems resulting in the release of cellular mediators and effector molecules, such as interleukin (IL)-6 and IL-8 and antimicrobial peptides, which help to eradicate the pathogen [19].

Little is known about the pathogenicity of *A. baumannii*. Moreover, the scarce reports on the virulence of *A. baumannii* are focused on one or a few strains only. The purpose of the present study was to obtain insight into the factors that might explain the clinical success of *A. baumannii*. To this aim, biofilm formation was investigated for a large set of well-described *A. baumannii* strains that differed in epidemicity and clonality. Next, biofilm formation by *A. baumannii* was compared to that of other *Acinetobacter* species, including *A.* gen. sp. 3 and 13TU, *A. calcoaceticus* and *A. junii*. For a subset of *A. baumannii* and *A. junii* strains, adherence to airway epithelial cells and induction of inflammatory cytokine production by these cells and cultured human macrophages was quantitated. Furthermore, the presence of pilus-like structures that may play a role in adherence and biofilm formation was assessed with scanning electron microscopy (SEM).

## Results

### Biofilm formation

**Figure 1:**
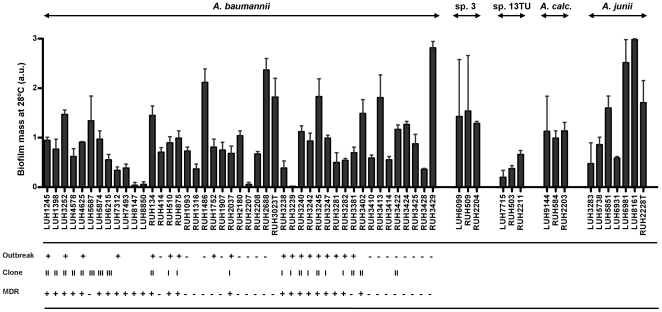
Biofilm formation by *Acinetobacter.* Biofilm formation after 24 h at 28°C for the clinically relevant *A. baumannii* (n = 45), *A.* gen. sp. 3 (n = 3) and *A.* gen. sp. 13TU (n = 3) and for the clinically less-relevant *A. calcoaceticus* (n = 3) and *A. junii* (n = 7). Data are expressed as mean biofilm mass (in arbitrary units (a.u.)) of three independent experiments; each performed in sixplicate. Outbreak-associated (+) or non-outbreak-associated (−) isolate. European clone I (I), II (II) or III (III) isolate. Multidrug resistant (MDR; +) or susceptible (−) isolate.

Biofilm formation on plastic at 28°C and 37°C was first investigated for a comprehensive set of *A. baumannii* strains. The results revealed a large variation in biofilm formation among *A. baumannii* isolates; the results at 28°C and 37°C did not differ (Fig. 1). There was no difference in the median biofilm size between strains from outbreaks (0.9; 0–1.8 a.u.) and those not assumed to be from outbreaks (0.8; 0.1–2.8 a.u.). Strains of European clone II (1.1; 0.6–1.8 a.u.) formed larger (p≤0.05) biofilms than strains of clone I (0.8; 0–1.0 a.u.), but not larger than strains of clone III (1.0; 0.6–1.3 a.u.). Multidrug resistant strains (0.8; 0–1.8 a.u.) did not form larger biofilms than susceptible strains (0.8; 0.1–2.8 a.u.). Furthermore, no association between biofilm formation and body site of isolation was found (data not shown).

Next, biofilm formation by *A. baumannii* strains was compared to that by strains of other *Acinetobacter* species. No significant differences in the median size of the biofilms at 28°C were found between clinically relevant species, i.e., *A. baumannii* (0.8; 0–2.8 a.u.), *A.* gen. sp. 3 (1.4; 1.3–1.5 a.u.) and *A.* gen. sp. 13TU (0.4; 0.2–0.7 a.u.), and the other *Acinetobacter* species, i.e., *A. calcoaceticus* (1.1; 1.0–1.1 a.u.) and *A. junii* (1.6; 0.5–3.0 a.u.). Similar results were obtained at 37°C except for strains of *A.* gen. sp. 13TU that formed smaller (p≤0.05) biofilms than all other species. Of note, the number of bacteria in the supernatants at the end of each experiment did not differ much between the different strains (data not shown).

### Adherence to human airway epithelial cells

For further comparison of clinically relevant to less-relevant *Acinetobacter* strains and species, experiments exploring the ability of strains to adhere to and induce cytokines by human cells were conducted with a subset of strains (further referred to as subset), including six strains of *A. baumannii* and six strains of *A. junii*, a species infrequently causing infections (Table 1). All selected strains were epidemiologically unrelated and were genotypically diverse as assessed by AFLP genomic fingerprinting.

**Table 1:** Characteristics of subset of *Acinetobacter* strains.

Strain	Origin		Year	Specimen	Clone	Outbreak*	Biofilm formation†		% Bacterial-associated cells‡		No. of bacteria per cell‡		IL-6 (ng/ml)¶		IL-8 (ng/ml)¶	
***A. baumannii***																
LUH1398	St. Zagora	(BG)	1993	Throat secr.	II		0.8	(0.5–1.1)	38	(32–65)	3	(3–3)	0.9	(0.0–3.9)	1.5	(0.4–4.8)
LUH7312	Leiden	(NL)	2001	Sputum		+	0.3	(0.3–0.4)	53	(38–76)	5	(3–6)	2.1	(0.1–14.5)	3.5	(0.8–12.8)
LUH7493	Leiden	(NL)	2001	Sputum			0.4	(0.3–0.5)	35	(16–46)	2	(2–3)	1.1	(0.0–3.6)	2.3	(0.1–4.8)
RUH875	Dordrecht	(NL)	1984	Urine	I	+	1.0	(0.8–1.1)	24	(17–45)	3	(2–3)	1.2	(0.0–5.6)	2.0	(0.3–4.6)
RUH3023T (ATCC19606)	Atlanta	(USA)	1965	Urine			1.9	(0.9–2.2)	19	(9–21)	2	(1–2)	0.6	(0.0–2.7)	1.0	(0.1–3.5)
RUH3239	London	(UK)	1985	Urine	I	+	0.0	(0.0–0.0)	6	(3–14)	1	(1–2)	1.0	(0.0–4.4)	1.5	(0.1–3.5)
***A. junii***																
LUH3283	Leiden	(NL)	1995	Blood			0.7	(0.0–0.8)	70	(62–83)	3	(3–4)	2.0	(0.1–11.9)	2.5	(0.8–10.0)
LUH5851	Leiden	(NL)	1999	Ear			1.6	(1.4–1.8)	31	(15–41)	2	(2–3)	2.0	(0.2–11.8)	3.3	(1.6–8.1)
LUH6931	Nottingham	(UK)	2000	Faeces			0.6	(0.6–0.6)	12	(9–13)	2	(2–2)	1.4	(0.0–7.4)	2.1	(0.4–8.7)
LUH6981	Leiden	(NL)	2000	Faeces			2.7	(2.6–3.0)	9	(5–29)	2	(1–2)	1.7	(0.1–12.3)	4.9	(1.5–11.5)
LUH8161	Leiden	(NL)	2002	Blood			3.0	(3.0–3.0)	15	(8–25)	3	(3–3)	3.1	(0.1–8.7)	3.8	(0.7–9.9)
RUH2228T (ATCC17908)	Heidelberg	(GER)	<1962	Urine			1.7	(1.1–1.8)	47	(45–48)	3	(2–3)	2.2	(0.1–13.1)	3.0	(0.8–9.4)

*Outbreak-associated (+) strain (i.e., common AFLP profile in >2 patients and with same time-space-origin).

†Biofilm formation on plastic after 24 h at 28°C. Results are expressed as median optical density values at 590 nm (ranges) of three independent experiments; each performed in sixplicate.

‡Adherence to human bronchial epithelial H292 cells after 1 h. Results are expressed as median percentage of H_292_ cells that is associated with bacteria and median number of bacteria per H_292_ cells (range) of two independent experiments; each performed in duplicate.

¶IL-6 and IL-8 levels in the culture supernatants of H_292_ cells, 24 h after exposure to bacterial strains. Results are expressed as median values (range) in ng/ml of three independent experiments; each performed in triplicate.

Adherence to human airway epithelial H_292_ cells varied widely among the *Acinetobacter* strains (Table 1). No significant difference in the percentage of H_292_ cells associated with bacteria was observed between *A. baumannii* (30; 7–55%) and *A. junii* (23; 11–70%). In addition, no difference in the number of *A. baumannii* and *A. junii* per positive H_292_ cell was seen; the median number of bacteria per H_292_ cell was 3 (range 1–5). Of note, differences in the inoculum (within the set range of 7×10^6^–4×10^7^) between the various experiments did not influence the outcome of the adherence assay. Furthermore, cell monolayers remained intact and the morphology of the cells was not affected by the bacteria (data not shown).

### Cytokine production by human airway epithelial cells in response to *Acinetobacter*

Pilot experiments demonstrated that 1×10^8^ CFU bacteria induced higher (p≤0.05) levels of the major cytokines IL-6 and IL-8 in H_292_ cells than 1×10^6^ and 1×10^7^ CFU did. Furthermore, stimulation with live bacteria resulted in higher (p≤0.05) IL-6 and IL-8 production by H_292_ cells than heat-inactivated bacteria did. Time-course experiments demonstrated that the levels of IL-6 and IL-8 were higher (p≤0.05) after 24 h than after 6 h stimulation. Therefore, further stimulation experiments were performed for 24 h with 1×10^8^ CFU live bacteria.

**Figure 2:**
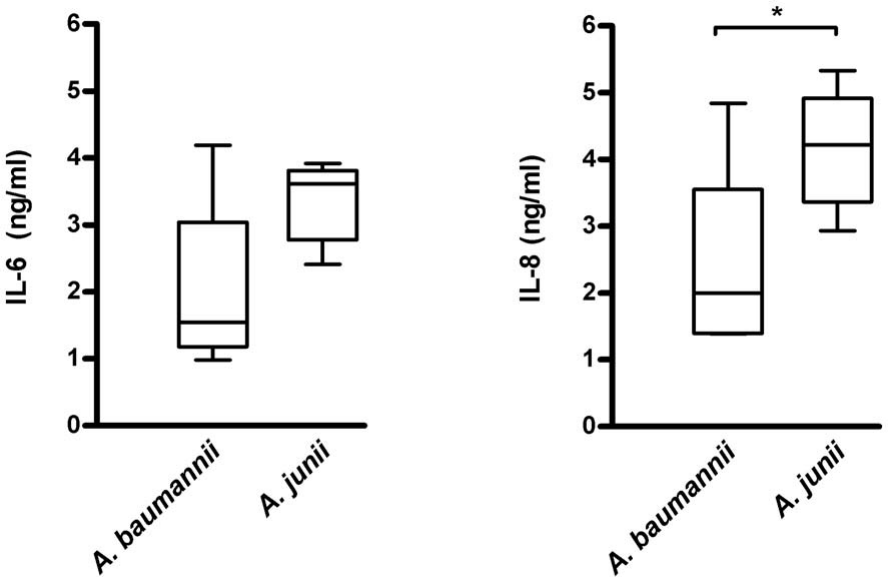
Cytokine production by human airway epithelial cells in response to *Acinetobacter*. Boxplots showing IL-6 and IL-8 production in ng/ml by H_292_ airway epithelial cells 24 h after stimulation with strains of *A. baumannii* (n = 6) and *A. junii* (n = 6). Boxes represent medians and second and third interquartiles, whiskers represent range of 6 strains. *significant (p≤0.05) difference between *A. baumannii* and *A. junii*.

Results revealed that H_292_ cells produced less (p≤0.05) IL-8 in response to *A. baumannii* strains (1.8; 1.0–3.5 ng/ml) than to *A. junii* strains (3.2; 2.1–4.9 ng/ml; Fig. 2). Interleukin-6 production was lower in response to *A. baumannii* strains (1.1; 0.6–2.1 ng/ml) than to strains of *A. junii* (2.0; 1.4–3.1 ng/ml; Fig. 2), without reaching statistical significance (p = 0.055). Of note, H_292_ cells stimulated with cytomix produced 2.3 (0.6–3.0) ng/ml IL-6 and 3.4 (1.4–4.1) ng/ml IL-8 and unstimulated cells produced 0.1 (0–0.1) ng/ml IL-6 and 0.2 (0.1–0.2) ng/ml IL-8. Cytokine induction did not vary widely among strains of each species, except for *A. baumannii* strain LUH7312 that induced significantly higher levels of IL-6 and IL-8 than the other *A. baumannii* strains. Stimulation of H_292_ cells with 7 additional *A. baumannii* strains that were epidemiologically unrelated and genotypically diverse also resulted in low IL-8 (1.3; 0.3–1.7 ng/ml) and IL-6 production (0.1; 0–0.2 ng/ml), demonstrating that, with the exception of strain LUH7312, *A. baumannii* strains induced significantly (p≤0.01) less IL-6 and IL-8 than *A. junii* strains.

Since live bacteria were used in this assay, there was a possibility of outgrowth that may have caused the difference in cytokine induction. However, the number of bacteria in the supernatants at the end of each experiment did not differ much between *A. baumannii* and *A. junii* (range 8×10^6^–7×10^7^ CFU/ml). Of note, cell monolayers remained intact and the morphology of the cells was not affected by the bacteria (data not shown).

Finally, preliminary experiments demonstrated that exposure of primary human bronchial epithelial cells of a single donor to six *A. baumannii* strains (in triplicate) resulted in lower (p≤0.05) levels of IL-8 (0.9; 0.5–3.6 ng/ml) than to six *A. junii* strains (4.5; 0.9–30.7 ng/ml). Cytomix induced 8.6 (7.4–11.5) ng/ml IL-8 in these cells. Of note, no IL-6 was produced by these primary cells upon exposure to these two *Acinetobacter* species or to cytomix.

### Cytokine production by cultured human macrophages in response to *Acinetobacter*

**Figure 3:**
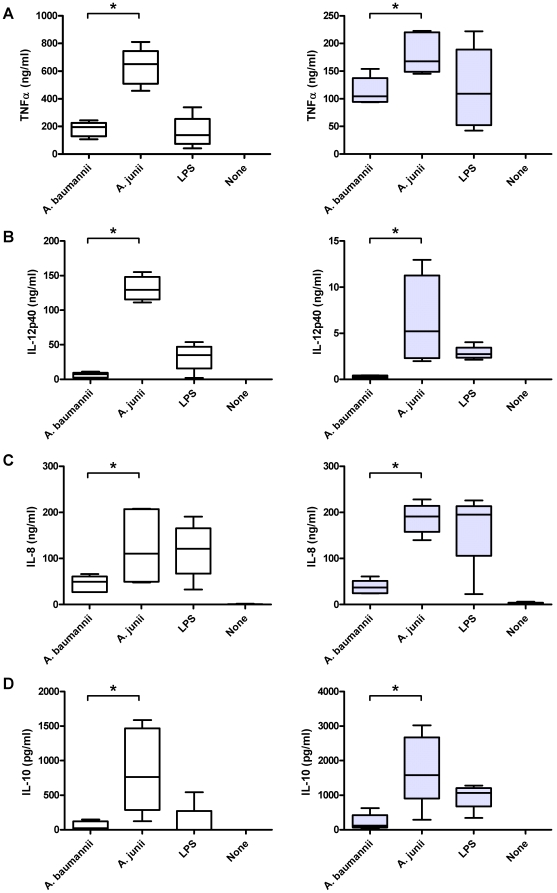
Cytokine production by human macrophages in response to *Acinetobacter*. Boxplots showing TNFα (A), IL-12p40 (B), IL-8 (C) and IL-10 (D) production by cultured human macrophages type 1 (white boxes) and 2 (gray boxes) 24 h after stimulation with strains of *A. baumannii* (n = 6), *A. junii* (n = 6), LPS and without stimulation (none). Boxes represent medians and second and third interquartiles, whiskers represent range of 6 strains (for *A. baumannii* and *A. junii*) or range of experiments with 4 different donors (for LPS and none). *significant (p≤0.05) difference between *A. baumannii* and *A. junii*.

In tissues, macrophages trigger an adequate innate immune response upon encountering pathogens. In this regulatory process, macrophages serve a dual purpose. Initially, they contribute to the elimination of pathogens and the elicitation of an inflammatory reaction. When the infection recedes due to removal of the pathogens, their function may shift toward resolution of inflammation and tissue repair. In line with this notion, we investigated cytokine production by two clearly distinct types of macrophages, i.e., pro-inflammatory macrophages (further referred to as type 1 macrophages), and macrophages with an anti-inflammatory/pro-angiogenic phenotype (type 2 macrophages) [20], upon exposure to *A. baumannii* and *A. junii* strains. Results revealed that macrophage type 1 produced significantly (p≤0.05) less tumor necrosis factor (TNF)α, IL-12p40, IL-10 and IL-8 in response to strains of *A. baumannii* [195 (108–244) ng/ml TNFα, 7 (2–11) ng/ml IL-12p40, 22 (0–149) pg/ml IL-10 and 49 (27–66) ng/ml IL-8] than to strains of *A. junii* [650 (458–812) ng/ml TNFα, 130 (111–155) ng/ml IL-12p40, 764 (126–1587) pg/ml IL-10 and 111 (48–208) ng/ml IL-8; Fig. 3]. *A. baumannii* strains also induced less (p≤0.05) inflammatory cytokines in macrophage type 2 than *A. junii* strains (Fig. 3). Of note, cell monolayers remained intact and the morphology of the cells was not affected by the bacteria (data not shown).

### Electron microscopy analysis of bacterial surface structures

**Figure 4:**
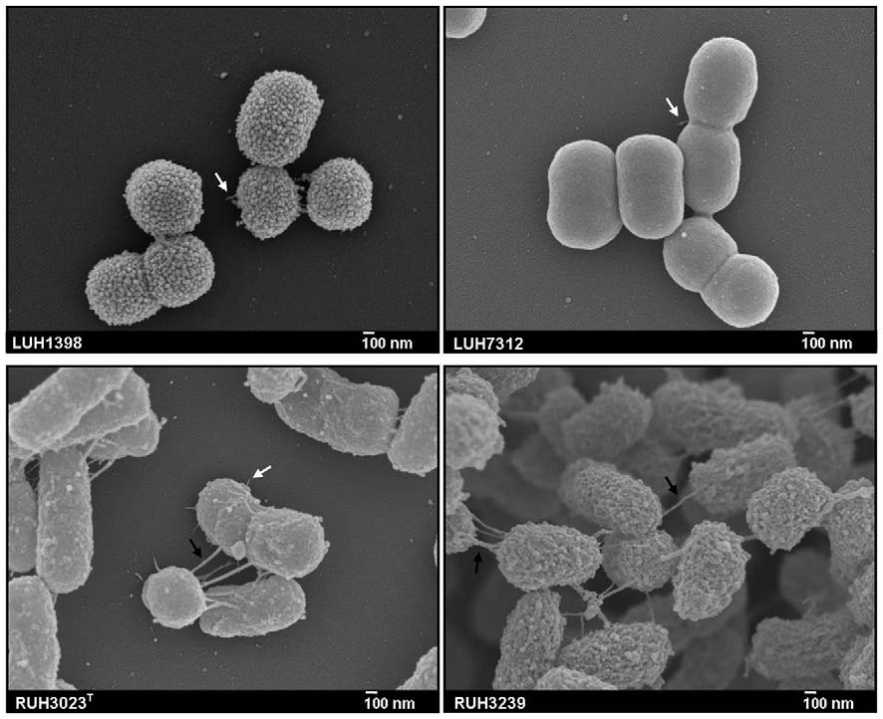
Scanning electron micrographs of *A. baumannii*. Scanning electron micrographs of *A. baumannii* strain LUH1398, LUH7312, RUH3023, and RUH3239. Black arrows indicate long cell extensions; white arrows indicate short pili-like structures. Magnification: 30,000×. Bars: 100 nm.

Pili have been described to be involved in biofilm formation, adherence and the induction of an immune response [21]. Therefore, we performed SEM to assess the presence of such surface structures on four *A. baumannii* strains that differed in their ability to form biofilm and adhere to human cells. SEM of bacteria cultured for 16 h at 37°C on blood agar plates revealed two types of cell appendages: short pilus-like structures and long extensions (Fig. 4). The latter varied in length, were irregularly distributed over the cell surface, and sometimes connected bacteria. The pilus-like structures were detected in *A. baumannii* strain LUH1398, a large biofilm former and highly adherent, in LUH7312, a small biofilm former and highly adherent, and RUH3023^T^, a large biofilm former but poorly adherent (Fig. 4, white arrows). Long cell extensions were seen in *A. baumannii* strain RUH3023^T^ and RUH3239, a small biofilm former and poorly adherent (Fig. 4, black arrows). In addition to these structures, there was a marked surface heterogeneity, from smooth (LUH7312) to pockmarked (LUH1398). In contrast to *A. baumannii* strain RUH3023^T^ and RUH3239, strain LUH1398 and LUH7312 formed only a few cell clusters with no more than four cells grouped together on the glass slides (data not shown). No difference in structural features was observed between strains cultured at 37°C and 30°C.

## Discussion

This study was undertaken to obtain some insight into the mechanisms underlying the clinical success of *A. baumannii*. The main conclusion from the present study is that strains of *A. baumannii* induced a poor inflammatory response in human cells, despite the finding that they adhered well to these cells. This conclusion is based on the following findings.

First, airway epithelial H_292_ cells in vitro produced less IL-6 and IL-8 in response to *A. baumannii* strains than to *A. junii* strains. Furthermore, cultured human macrophages produced less TNFα, IL-12p40, IL-8 and IL-10 in response to *A. baumannii* strains than to *A. junii* strains. In agreement with our in vitro data, Qiu et al documented that the high susceptibility of A/J mice to *A. baumannii* ATCC17961 infection was associated with a reduced local pro-inflammatory response and reduced elimination of bacteria from the lungs [22]. Knapp et al showed in an in vivo model that *A. baumannii* strain RUH2037 induced the release of pro-inflammatory cytokines and chemokines resulting in clearance of bacteria from the lungs of experimentally infected mice [23]. Although study-design and outcome were distinct from our study, the findings emphasize the importance of inflammatory cytokines for clearing of *A. baumannii*. It is furthermore of note that our finding that clinically relevant strains induced a weak immune response in vitro has also been reported for *Haemophilus influenzae* [24].

There was a wide variation in biofilm formation among a large set of well-described *A. baumannii* strains. Although epidemic strains did not form larger biofilms than sporadic strains, it appeared that strains of European clone II formed larger biofilms than strains of clone I. It is of note that clone II, which was less frequently involved in outbreaks during the 1990s than clone I, is now emerging in several European countries with many strains being carbapenem resistant [25], [26]. Further to *A. baumannii*, the intra-strain variation in biofilm formation of *A.* gen. sp. 3 and 13TU and of the clinically less-relevant species *A. calcoaceticus* and *A. junii* was also considerable. Interestingly, there was no difference between these species, except for *A.* gen. sp. 13TU that formed small biofilms.

Many of the strains used for the current biofilm experiments had previously been used to investigate adherence to H_292_ airway epithelial cells [18]. In that study, it was shown that adherence to airway epithelial cells varied considerably among strains of *A. baumannii*, while strains of clone II had higher adherence values than those of clone I [18]. Likewise, the present study showed a considerable intra-species variation in adherence to human airway epithelial cells but no difference between *A. baumannii* and *A. junii* strains. Biofilm formation on plastic was not correlated to adherence to human cells, indicating that different mechanisms are involved in these colonization processes. Furthermore, the ability to form biofilm on plastic and the capacity to adhere to human cells was not always accompanied by the presence of pilus-like cell surface structures and long cell extensions. Taken together, it seems that biofilm formation and adherence to human cells is strain- and not species-specific. Thus, these features do not solely explain the success of *A. baumannii* in the susceptible host.

In summary, biofilm formation and adherence to airway epithelial cells did not differ between clinically relevant and less-relevant *Acinetobacter* strains and species. However, there was a difference in the production of inflammatory cytokines by airway epithelial cells and macrophages between *A. baumannii* and *A. junii.* This may be a first clue to explain the difference in clinical behavior between *A. baumannii* and *A. junii*. We hypothesize that *A. baumannii* may survive and persist in the airways of patients and cause disease at least in part by inducing a weak inflammatory response.

## Materials and Methods

### Bacterial strains, culture conditions and antimicrobial susceptibility testing

Forty-five *A. baumannii*, 3 *A.* gen. sp. 3, 3 *A.* gen. sp. 13TU, 3 *A. calcoaceticus*, and 7 *A. junii* isolates, were selected from the Leiden University Hospital *Acinetobacter* collection for this study. Of the *A. baumannii* strains, 18 were from outbreaks, 16 presumably not from outbreaks on basis of time-space-origin, and 11 of which the association with an outbreak was unknown. Eight of the *A. baumannii* strains belonged to European clone I, 11 to clone II, and 3 to clone III. All isolates had previously been identified to species by one or more validated genotypic identification methods [27], [28], [29]. Bacteria were preserved for prolonged periods in nutrient broth supplemented with 20% (vol/vol) glycerol at -80°C. Prior to each experiment, inocula from frozen cultures were grown overnight at 30°C [30] on sheep blood agar plates (BioMerieux, Boxtel, the Netherlands). For experiments, fresh subcultures were made either under these conditions or in Luria-Bertani (LB) medium. Susceptibility to antimicrobial agents was determined by disc diffusion according to CLSI recommendations [31]. Strains resistant to more than two of the following drug classes were defined as multidrug resistant: cephalosporins, carbapenems, ampicillin-sulbactam, quinolones, aminoglycosides.

### Biofilm formation

Biofilm formation in 96-wells polyvinylchloride microtiter plates (Falcon, BD, Breda, the Netherlands) was assayed as described [32]. Briefly, bacteria from an overnight culture in LB medium were suspended to 1×10^6^ CFU/ml as calculated from the absorbance of a suspension at 600 nm. Five µl of this suspension was inoculated in 100 µl of M63 medium consisting of KH_2_PO_4_ (12 g/l), K_2_HPO_4_ (7 g/l), (NH_4_)_2_SO_4_ (2 g/l), glucose (0.2% w/v), MgSO_4_ (1 mM) and casaminoacids (0.5% w/v). After 24 h incubation at 28°C and 37°C, wells were washed and biofilms attached to the wells were stained with crystal violet (1% w/v). The optical density at 590 nm, expressed in arbitrary units (a.u.), was taken as a quantitative measure of biofilm mass. To determine the bacterial concentration after 24 h, serial dilutions of the supernatants were made in PBS and plated onto blood agar.

### Adherence to human airway epithelial cells

Adherence of bacteria to human bronchial epithelial H_292_ cells (ATCC CRL-1848, Manassas, VA, USA) was determined as described [18]. Briefly, H_292_ cells were incubated for 1 h at 37°C with 1×10^7^ (range 7×10^6^–4×10^7^) CFU of an overnight culture on blood agar. Bacterial adherence to H_292_ cells was quantified by light microscopy using two parameters: (i) percentage of epithelial cells associated with at least one bacterium; and (ii) average number of bacteria per epithelial cell [18].

### Cytokine induction in human airway epithelial cells

Cytokine production by airway epithelial cells in response to bacteria was determined as described previously [33] with minor modifications. In short, bacteria were cultured overnight at 30°C on blood agar and suspended in RPMI-1640 to a concentration of 5×10^8^ CFU/ml as assessed spectrophotometrically. Approximately 2×10^5^ H_292_ cells were cultured in 0.5 ml of RPMI-1640 supplemented with 2 mM L-glutamine, 50 µg/ml streptomycin, 1000 U sodium penicillin G, and 10% (vol/vol) heat-inactivated foetal calf serum (FCS_i_), further referred to as culture medium, in 24-wells plates. At 85–90% confluency, H_292_ cells were washed with prewarmed sterile phosphate buffered saline (PBS; pH 7.4) and incubated at 37°C and 5% CO_2_ in culture medium without FCSi for 24 h. Subsequently, cells were washed with prewarmed PBS and incubated for 1 h at 37°C and 5% CO_2_ with 1×10^6^, 1×10^7^, or 1×10^8^ CFU live or heat-inactivated (by 1 h incubation at 100°C) bacteria. H_292_ cells were washed five times with prewarmed PBS to remove non-adherent bacteria and fresh RPMI-1640 with 2 mM L-glutamine was added. After additional 5 or 23 h incubation at 37°C, supernatants were collected and stored at −20°C. After 24 h, bacterial CFU count in the supernatants was determined. In each experiment, RPMI-1640 with 2 mM L-glutamine alone was added to the cells to determine background values. A mixture of cytokines (100 ng/ml TNFα, 20 ng/ml IL-1β and 10 ng/ml IFNγ; all from Biosource, Nivelles, Belgium) with 10 ng/ml rough type lipopolysaccharide (*Escherichia coli* J5, Sigma-Aldrich, Zwijndrecht, the Netherlands), further referred to as cytomix, was added to the cells as a positive control.

### Cytokine induction in cultured human macrophages

Buffy coats from healthy human donors were purchased from Sanquin bloodbank, Amsterdam, the Netherlands, upon written consent with regard to scientific use. The current study did not require approval from an ethics committee according to the Dutch Medical Research Involving Human Subjects Act. Monocytes were isolated from buffycoats by Ficoll amidotrizoate density centrifugation and magnetic sorting using anti-CD14-coated beads (Miltenyi Biotec, Auburn, CA, USA) according to the manufacturer's instructions. Monocytes were resuspended in culture medium and cultured at a concentration of 2×10^5^ cells/ml in wells of a 24-wells plate at 37°C, 5% CO_2_ in the presence of either 5 ng/ml recombinant human granulocyte–macrophage colony-stimulating factor (Biosource International, Camarillo, CA, USA) to induce differentiation of monocytes into macrophage type 1 or in the presence of 50 ng/ml recombinant human macrophage colony-stimulating factor (R&D Systems, Minneapolis, MN, USA) for type 2 macrophages [20]. After three days, 0.5 ml of fresh culture medium was added. On the sixth day following isolation, cells were washed once in PBS and 0.5 ml RPMI-1640 with 2 mM L-glutamine was added. Bacteria were cultured overnight at 30°C on blood agar and suspended in PBS to a concentration of 5×10^7^ CFU/ml as assessed spectrophotometrically. Cells were stimulated with 1×10^7^ CFU of live bacteria for 24 h, after which supernatants were collected and stored at −20°C. In each experiment, PBS alone was added to the cells to determine background. Lipopolysaccharide (100 ng/ml, *Escherichia coli* J5, Sigma-Aldrich) was added to the cells as a positive control.

### Determination of levels of inflammatory mediators

The levels of IL-6, IL-8, TNFα, IL-12p40 and IL-10 in culture supernatants were determined by ELISA (Biosource) according to the manufacturer's instructions. The lower limit of detection was 15 pg/ml for IL-6, 7 pg/ml for IL-8, and 25 pg/ml for TNFα, IL-12p40 and IL-10.

### Electron microscopy analysis of bacterial surface structures

For SEM, bacteria from an overnight culture on blood agar at 30°C were suspended in PBS and fixed for 1 h at room temperature with 1.5% (w/v) glutaraldehyde in 0.1 M sodium cacodylate buffer (1∶1). Fixed bacteria were transferred to poly-L-lysine-coated glass slides. After 1 h incubation at room temperature, bacteria were fixed to the slides with 1.5% glutaraldehyde for 30 min at room temperature. Thereafter, slides were washed twice in PBS, dehydrated through a series of graded ethanol, critical-point-dried, and coated with a layer of palladium-gold. Bacteria were examined using a JEOL JSM-6700F field emission scanning electron microscope.

### Statistical analysis

Results are expressed as medians and ranges. The Kruskal-Wallis one-way analysis of variance and the Wilcoxon rank sum test were used to evaluate differences in distribution. Spearman rank correlation coefficients were calculated to evaluate possible associations between epidemicity and adherence to epithelial cells and biofilm formation on plastic. P values of ≤0.05 were considered significant.
